# Abstracts najaarsvergadering NVU, 5 november 2021

**DOI:** 10.1007/s13629-021-9004-8

**Published:** 2021-10-22

**Authors:** Bohn Stafleu van Loghum

**Affiliations:** http://www.bsl.nl

## Abstract

Patiënten met prostaatkanker (PCa) kunnen geselecteerd worden voor zenuwsparende robotgeassisteerde radicale prostatectomie (RARP) middels voorspellingsmodellen voor extraprostatische extensie (EPE). In 2019 hebben Soeterik et al. een *easy-to-use* voorspellingsmodel met MRI-tumorstadium ontwikkeld en extern gevalideerd voor zijdespecifieke EPE. In deze studie werd dit model getest in een cohort van patiënten die systematische biopten, MRI-gerichte biopten of beide ondergingen.

## 1. Externe validatie van een voorspellingsmodel voor zijdespecifieke extraprostatische extensie bij gelokaliseerd prostaatkanker: het Soeteriknomogram

H. Veerman, M.W. Heymans, P.J. van Leeuwen, A.N. Vis en H.G. van der Poel

Nederlands Kanker Instituut – Antoni van Leeuwenhoek, Amsterdam

### Introductie

Patiënten met prostaatkanker (PCa) kunnen geselecteerd worden voor zenuwsparende robotgeassisteerde radicale prostatectomie (RARP) middels voorspellingsmodellen voor extraprostatische extensie (EPE). In 2019 hebben Soeterik et al. een *easy-to-use* voorspellingsmodel met MRI-tumorstadium ontwikkeld en extern gevalideerd voor zijdespecifieke EPE. In deze studie werd dit model getest in een cohort van patiënten die systematische biopten, MRI-gerichte biopten of beide ondergingen.

## Materiaal en methoden

Een prospectief verzameld cohort van 1170 opeenvolgende patiënten die RARP ondergingen in twee hoogvolumecentra tussen 2018 en augustus 2021 werd retrospectief onderzocht. Alle patiënten ondergingen een MRI-scan voor de operatie. De diagnose PCa werd gesteld met systematische biopten, MRI-targeted biopten of beide. Met het Soeterik-nomogram werd de zijdespecifieke kans op EPE berekend voor elke prostaathelft met complete data (PSA-densiteit, biopt Gleason-scores, radiologisch tumorstadium). Modeldiscriminatie en -kalibratie werden onderzocht middels de *area under the curve* (AUC), *calibration-in-the-large* en calibratiecurves.

## Resultaten

Pathologische EPE werd vastgesteld in 30% van de prostaathelften. De gemiddelde voorspelde kans op EPE was ook 30%. De onderscheidende waarde van het model was goed (AUC 80,4% (IQR 78,4%-82,3%)). De voorspelde kans kwam goed overeen met het geobserveerde percentage van EPE met een intercept van -0,02 en een helling van 1,053. Er was onderschatting van het geobserveerde percentage EPE vanaf een voorspelde kans van 70%. De klinische consequentie van deze onderschatting is discutabel aangezien zenuwsparende chirurgie doorgaans wordt afgeraden bij deze drempelwaarde in verband met de kans op positieve snijvlakken. Zie figuur 1.1.

**Figure:**
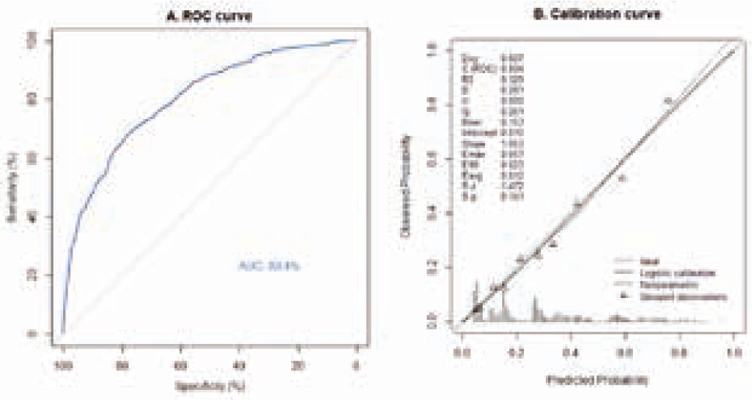
**Figuur 1.1**

## Conclusie

Het Soeterik-nomogram heeft een goede fit op een grote groep Nederlandse patiënten. Het nomogram kan binnen Nederland goed gebruikt worden om patiënten te selecteren voor zenuwsparende prostatectomie.

## 2. Ontwikkeling van een nieuw nomogram voor de preoperatieve predictie van pelviene lymfekliermetastasen, inclusief de bevindingen op MRI en PSMA PET

D. Meijer, A.N. Vis, M.J. Roberts, P.M. van de Ven, H.G. van der Poel, M.L. Donswijk, T.N. Boellaard, I.G. Schoots, D.E. Oprea-Lager en P.J. van Leeuwen

Amsterdam Universitair Medische Centra, locatie VUmc, Amsterdam

### Introductie

Het preoperatief voorspellen van de kans op pelviene lymfe-kliermetastasen (pN1-ziekte) is cruciaal om patiënten te selecteren die in aanmerking komen voor een uitgebreide pelviene lymfeklierdissectie (ePLND), ten tijde van de radicale prostatectomie (RALP). Het doel van deze studie was een nieuw prognostisch model te ontwikkelen om de kans op pN1-ziekte te voorspellen bij patiënten met gelokaliseerd prostaatkanker, bestaande uit klinische en histologische parameters, alsmede de uitkomsten van preoperatieve beeldvormende technieken, zoals de MRI- en de PSMA PET-scan.

### Materiaal en methoden

Alle 680 patiënten die een PSMA PET- en MRI-scan ondergingen, voorafgaand aan de RALP met ePLND werden geïncludeerd. Door middel van een logistische regressieanalyse werd een prognostisch model ontwikkeld. De voorspellende waarde van dit nieuwe nomogram werd onderzocht met behulp van de *area under the curve* (AUC). Deze werd vergeleken met de prognostische waarde van bestaande nomogrammen, zoals het MSKCC- en het Briganti-nomogram.

### Resultaten

In totaal hadden 175/680 patiënten (26%) pelviene lymfekliermetastasen bij histopathologische evaluatie. Het nieuwe nomogram bestond uit de initiële PSA-waarde, radiologisch T-stadium op MRI, hoogste biopsie Grade Group (GG), biopsietechniek (MRI-targeted versus systematisch), percentage systematische biopten met klinisch significant prostaatkanker (GG ≥ 2) en bevindingen op de PSMA PET-scan. De AUC voor het voorspellen van pN-ziekte was 0,80 (95%-BI 0,74-0,82) met het nieuwe model, vergeleken met 0,69 met zowel het MSKCC-nomogram en het Briganti-nomogram. Zie figuur 2.1.

**Figure:**
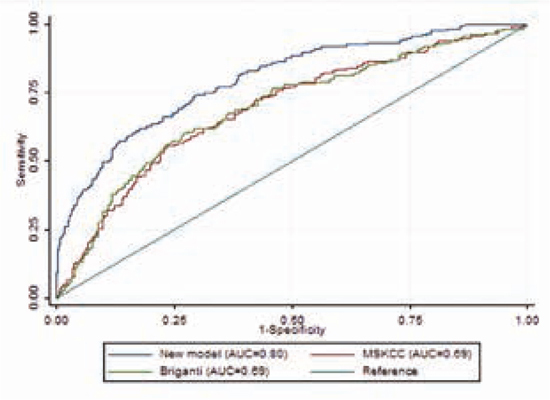
**Figuur 2.1**

### Conclusies

Het nieuwe nomogram voor het voorspellen van pN1-ziekte is ontwikkeld in patiënten met gelokaliseerd prostaatkanker, en bestaat uit klinische en histologische parameters, aangevuld met de resultaten van de MRI-scan en de PSMA PET-scan. Het nieuwe nomogram blijkt superieur aan alle bestaande nomogrammen en zorgt voor het (terecht) nalaten van een pelviene lymfeklierdissectie bij een substantieel deel van de patiënten.

## 3. Intravesicale botulinetoxine-injecties bij uitbehandelde kinderen met een niet-neurogene overactieve blaas

A.P. Lambregts, A.J. Nieuwhof-Leppink, A.J. Klijn en R.P.J. Schroeder

Universitair Medisch Centrum Utrecht, Utrecht

### Introductie

Een overactieve blaas met urine-incontinentie bij kinderen heeft een grote impact op de dagelijkse activiteiten en kwaliteit van leven. In sommige gevallen reageert een overactieve blaas niet op urotherapie en anticholinergica. Vanwege de succesvolle uitkomsten bij de behandeling van een neurogene blaas, bieden intravesicale botulinetoxineinjecties een mogelijke oplossing bij kinderen die ongevoelig zijn voor therapie. In deze studie analyseren we de uitkomsten van botulinetoxine-injecties op blaasvolume en incontinentie bij kinderen met een overactieve blaas.

### Materiaal en methoden

Van 50 kinderen die waren gediagnosticeerd met een uitbehandelde niet-neurogene overactieve blaas die botulinetoxine-injecties ontvingen werden de dossiers retrospectief geanalyseerd. Het functionele blaasvolume wordt uitgedrukt als percentage van de verwachte blaascapaciteit voor de leeftijd. Respons wordt beschreven als verbetering in urge-incontinentie na botulinetoxine-injecties. Er werd een multivariate analyse uitgevoerd om voorspellers aan te tonen voor de respons.

### Resultaten

Er werden 50 kinderen geïncludeerd met een mediane leeftijd 9,9 jaar met een man-vrouwratio van 1:4. Op de korte termijn (< 6 maanden) werd een significante groei van het functionele blaasvolume gezien met een mediaan van 52,9 naar 70% (*p =* 0,000). 72% van de kinderen liet een verbetering in urinecontinentie zien op de korte termijn en 46% op de lange termijn (> 6 maanden). Het mannelijk geslacht en een klein uitgangsblaasvolume voorspelden een goede uitkomst op continentie op de lange termijn. De meest voorkomende complicatie was een urineweginfectie, die optrad bij zes kinderen (12%). Zie figuur 3.1.

**Figure:**
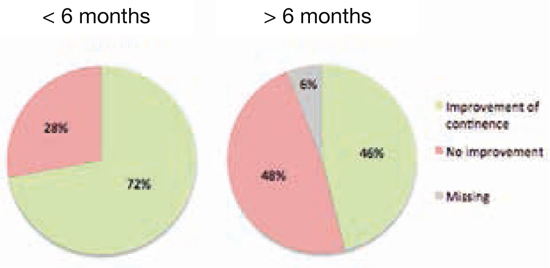
**Figuur 3.1**

### Conclusie

Botulinetoxine-injecties bij kinderen met een overactieve blaas zijn veilig en effectief ter vergroting van het blaasvolume en vermindering van symptomen, met name in de eerste zes maanden na de injectie.

## 4. Fosfomycine versus ciprofloxacine als orale uitbehandeling voor *Escherichia Coli*gecompliceerde urineweginfecties bij vrouwen: een gerandomiseerde, placebogecontroleerde, dubbelblinde, multicenter trial

M.M. Hobijn, M.E.M. van ’t Hof, E. L. Koldewijn, M.J.M. Bonten, T. ten Doesschate, mede namens het Forecast-studieteam Catharina Ziekenhuis Eindhoven

### Introductie

Voor de uitbehandeling van gecompliceerde urineweginfecties met een *Escherichia coli* bij vrouwen wordt veelal gekozen voor een breedspectrumantibioticum. Het doel van deze studie was om aan te tonen dat fosfomycine non-inferieur is aan ciprofloxacine voor de uitbehandeling van deze infecties.

### Materiaal en methoden

Een dubbelblind gerandomiseerde, placebogecontroleerde studie werd uitgevoerd in 15 Nederlandse ziekenhuizen. Volwassen vrouwen ontvingen 2-5 dagen empirische intraveneuze antibiotica voor een *E. coli* gecompliceerde urineweginfectie. Zij werden daarna uitbehandeld met eenmaal daags 3 g fosfomycine of tweemaal daags 500 mg ciprofloxacine, met een totale behandelduur van 10 dagen. Voor de primaire uitkomstmaat genezing (afname klinische en systemische symptomen zonder noodzaak tot verlenging van de antibioticakuur) op dag 6-10 na behandeling werd een non-inferieure marge van 10% gekozen. De studie was geregistreerd in Trialregister.nl (NTR6449).

### Resultaten

Na inclusie van 97 patiënten tussen 2017 en 2020, eindigde de studie voortijdig vanwege de COVID-19-pandemie. 50% van de patiënten hadden een bacteriemie. De primaire uitkomstmaat werd bereikt bij 36/48 patiënten in de fosfomycinegroep en 30/46 patiënten in de ciprofloxacinegroep. Secundaire uitkomstmaten waren microbiologische genezing (negatieve urinekweek) op dag 6-10, klinische genezing op dag 30-35 en bijwerkingen van de therapie. Zie figuur 4.1.

### Conclusie

Fosfomycine is non-inferieur aan ciprofloxacine voor de uitbehandeling van gecompliceerde urineweginfecties met *E. coli* bij vrouwen. Fosfomycinegebruik was geassocieerd met meer gastro-intestinale bijwerkingen.

## 5. Praktijkvariatie in de behandeling van hydrocele bij volwassenen: een multinationaal onderzoek

L.P.W. Witte, M.T. Forss, K. Bolsunovskyi, T.P. Kilpeläinen, Y. Lee, Y. Aoki, S. Gudjonsson, F. Hervé, P. Järvinen, S. Malde, J. Sairanen, L. Sander, G.H. Guyatt en K.A.O. Tikkinen

### Introductie

Ondanks de hoge incidentie van hydroceles bij volwassenen, hebben urologische beroepsverenigingen geen formele richtlijnen voor de behandeling hiervan. Ons doel was om internationale praktijkvariatie in de behandeling van een hydrocele bij volwassenen in kaart te brengen. Postoperatieve complicaties komen vaak voor na hydrocelectomie en recidieven komen vaak voor na aspiratie (met of zonder sclerotherapie).

### Materiaal en methoden

We hebben een internationaal onderzoek uitgevoerd naar de behandeling van een hydrocele onder urologen in België, Denemarken, Finland, IJsland, Japan en Nederland van september 2020 tot december 2020. We identificeerden urologen uit de registers van de landelijke urologische beroepsverenigingen en selecteerden willekeurig 170 urologen en urologen in opleiding uit elk deelnemend land (in IJsland hebben we alle urologen ondervraagd). Behalve in Finland (half e-mail en half post) en Japan (postenquête), vulden alle deelnemers een e-mailenquête in.

**Table:** **Tabel 4.1**

	**Fosfomycine** (*n* = 48)	**Ciprofloxacine** (*n* = 49)	**Risicoverschil** (95%-BI)
**6-10 dagen na behandeling**			
klinische genezing	36/48 (75,0%)	30/46 (65,2%)	9,6% (-8,8-8,0)
microbiologische genezing	29/37 (78,4%)	33/35 (94,3%)	-16,2% (-32,7-0,0)
**30-35 dagen na behandeling**			
klinische genezing	35/47 (74,5%)	33/44 (75,0%)	0,4% (-18,4-17,6)
algemene bijwerkingen	35/48 (72,9%)	32/46 (69,6%)	3,3% (-15,0-21,6)
gastro-intestinale bijwerkingen	25/48 (52,1%)	14/46 (30,4%)	20,8 (1,6-40)

### Resultaten

Van de 864 gecontacteerde urologen deden er 437 (51%) mee. Van de respondenten was 28% vrouw en 19% uroloog in opleiding en had 52% zowel hydrocelectomieën, als aspiraties uitgevoerd. In België (83%), Denemarken (55%) en Nederland (75%), voerden urologen het vaakst hydrocelectomieën uit, terwijl in Finland (84%), Japan (61%) en IJsland (91%) de meeste urologen zowel hydrocelectomieën als aspiraties uitvoerden. Urologen gaven de voorkeur aan een hydrocelectomie voor een grote hydrocele (80% vs. 38% voor kleine), jongere patiënten (67% voor patiënten < 50 jaar vs. 42% voor 70 jaar of meer), patiënten met weinig of geen comorbiditeit (65% vs. 24% voor patiënten met multipele comorbiditeit) en patiënten zonder bloedverdunners (55% vs. 37% voor patiënten met bloedverdunners). Zie figuur 5.1.

**Figure:**
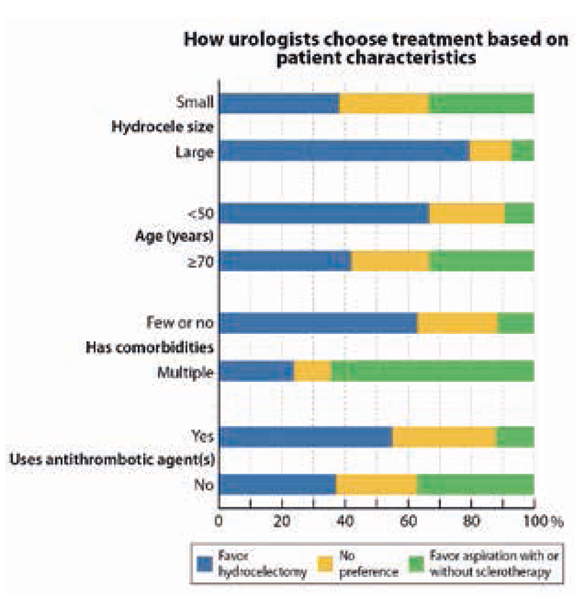
**Figuur 5.1**

### Conclusie

We vonden een grote variatie in de klinische praktijk met betrekking tot de behandeling van een hydrocele bij volwassenen binnen en tussen landen. Toekomstige studies en richtlijnen zijn nodig om deze variatie te verminderen en de behandeling van een hydrocele te standaardiseren wereldwijd.

## 6. Diagnostiek en therapie van torsio testis: houdt iedereen zich aan de richtlijn?

T. van Doeveren, B.K. Somani en S.M. Haensel

Franciscus Gasthuis, Rotterdam

### Introductie

Torsio testis is een acute diagnose en de meest voorkomende oorzaak van irreversibele testiculaire ischemie bij kinderen en adolescenten. Snelle diagnostiek en een juiste behandeling kan blijvende schade voorkomen. De EAU richtlijn *Pediatric Urology 2021* voorziet in adviezen, maar er blijven veel onduidelijke elementen bestaan over diagnostiek en behandeling. Om navolging van de richtlijn in kaart te brengen en internationale routines te vergelijken, werd een online vragenlijst afgenomen.

### Methode

De online vragenlijst werd via de NVU en een internationaal netwerk verspreid onder urologen en a(n)ios (hierna *respondenten*). De vragenlijst bestond uit 18 vragen over diagnostiek, therapie en demografie en werd ingeleid met een casus.

### Resultaten

De vragenlijst werd 303 maal ingevuld; 70,6% daarvan door respondenten in Nederland. Echografie wordt altijd verricht door 64,7% van de respondenten. Een manuele detorsie, die in de richtlijn wordt aanbevolen, wordt op de SEH slechts door 38% van de respondenten verricht. Een detorsie wordt als succesvol gezien indien de pijn is verdwenen (28,4%) en 30,4% controleert dit met echo-Doppler. Torsio testis wordt door 76,5% van de respondenten gezien als een indicatie voor spoedchirurgie; 57% verricht een Winkelmann-plastiek. Fixatie van de getordeerde testis wordt door 77,2% verricht, waarbij 29,4% aangeeft de contralaterale testis alleen te fixeren bij een bewezen torsie. Opvallend is dat 28,0% van de Nederlandse respondenten een torsio testis niet als spoedoperatie behandelt, tegen slechts 12,4% internationaal. Verder blijkt 31,4% van de Nederlandse respondenten de testis niet te fixeren; tegenover 3,4% van de buitenlandse urologen. Zie tabel 6.1.

**Tabel 6.1:** Diagnostische, therapeutische en chirurgische karakteristieken (*n* = 303)

	**Nederland**	**Internationaal**	**Gehele cohort**	
	*n* (%)	*n* (%)	*n* (%)	*p*-waarde
**Diagnostiek**				
Gebruik scrotale echografie				
ja	136 (61,6)	60 (67,4)	196 (64,7)	< 0,001
alleen bij twijfel	76 (35,5)	20 (22,5)	96 (31,7)	
nooit	2 (0,9)	9 (10,1)	11 (3,6)	
**Therapie**				
Manuele detorsie op SEH				
ja, met pijnstilling	0 (0,0)	3 (3,4)	3 (1,0)	< 0,001
ja, zonder pijnstilling	93 (43,5)	19 (21,3)	112 (37,0)	
afhankelijk van beschikbaarheid ok	49 (22,9)	28 (31,5)	77 (25,4)	
nee, nooit	72 (33,6)	39 (43,8)	111 (36,6)	
Succesvolle detorsie?				
indien pijn volledig weg is	74 (34,6)	12 (13,5)	86 (28,4)	0,003
bij goede dopplersignalen echo	59 (27,5)	33 (37,1)	92 (30,4)	
testes in dezelfde positie	7 (3,3)	3 (3,4)	10 (3,3)	
manuele detorsie wordt niet uitgevoerd	74 (34,6)	41 (46,0)	115 (38,0)	
Indicatie voor spoedoperatie?				
ja, altijd	107 (50,0)	57 (64,0)	164 (54,1)	0,011
ja, ook na succesvolle detorsie	47 (22,0)	21 (23,6)	68 (22,4)	
nee, liever niet	60 (28,0)	11 (12,4)	71 (23,4)	
Timing operatie				
geen detorsie, direct OK	73 (34,1)	42 (47,3)	115 (38,0)	0,018
zo snel mogelijk na detorsie	50 (23,4)	26 (29,2)	76 (25,1)	
binnen 24 uur	9 (4,2)	6 (6,7)	15 (5,0)	
binnen 72 uur	10 (4,7)	2 (2,3)	12 (4,0)	
alleen bij een recidief	4 (1,9)	1 (1,1)	5 (1,7)	
eerste mogelijkheid reguliere planning	68 (31,7)	12 (13,4)	80 (26,4)	
**Chirurgie**				
Locatie incisie				
mediaal in de raphe	176 (82,2)	53 (59,6)	229 (75,6)	< 0,001
lateraal, parallel aan de raphe	35 (16,4)	31 (34,8)	66 (21,8)	
inguïnaal plus scrotaal	1 (0,5)	3 (3,4)	4 (1,3)	
inguïnaal	2 (0,9)	2 (2,3)	4 (1,3)	
Openen van de tunica vaginalis				
ja, met Winkelmann-plastiek	149 (69,6)	32 (36,0)	181 (59,7)	< 0,001
ja, zonder Winkelmann-plastiek	58 (27,1)	50 (56,1)	108 (35,6)	
nee, meestal niet	7 (3,3)	7 (7,9)	14 (4,6)	
Fixatie van de testis				
testis wordt niet gefixeerd	67 (31,3)	3 (3,4)	70 (23,1)	< 0,001
1-punts fixatie	8 (3,7)	14 (15,7)	22 (7,3)	
2-punts fixatie	60 (28,0)	35 (39,3)	95 (31,4)	
3-punts fixatie	79 (37,0)	37 (41,6)	116 (38,3)	
Fixatie van de contralaterale testis				
Ja, altijd	144 (67,3)	51 (57,3)	195 (64,4)	0,047
Alleen na een bewezen torsie	61 (28,5)	28 (31,5)	89 (29,4)	
Nee, nooit	9 (4,2)	10 (11,2)	19 (6,3)	

### Conclusie

De respondenten volgen in de diagnostiek en behandeling van torsio testis meestal de EAU-richtlijn. Wel blijkt er sprake van uiteenlopende meningen over manuele detorsie, gebruik van echografie in de diagnostiek, de timing van operatie en het fixeren van de contralaterale testis. Gezien het belang van de juiste behandeling is het belangrijk om de richtlijn op sommige punten aan te passen en uit te breiden, en deze vervolgens zo goed mogelijk op te volgen voor een eensgezind beleid.

## 7. Het patiëntentraject van mannen met een chronisch urineresidu: behandelingen en complicaties

B.J. Bos, N.A.M. van Merode, M.S. Steffens en L.P.W. Witte Isala Ziekenhuis, Zwolle

### Introductie

Er zijn verschillende behandelopties voor mannen met klachten van een chronische urineresidu (CUR). Wij hebben in kaart gebracht welke behandelingen patiënten ondergingen en wat de gerelateerde complicaties waren.

### Materiaal en methode

Voor deze *single-center*, retrospectieve studie werden alle mannen geïncludeerd met klachten van een niet-neurogeen CUR van > 150 ml gedurende ten minste vier maanden tussen 2014 en 1 september 2020.

## Resultaten

In totaal werden 177 mannen geïncludeerd. De mediane leeftijd was 77 (44-94) jaar. Het mediane residu na mictie was 158 (0,0-1200) ml. De gemiddelde IPSS was 17 ± 8 en de IPSS-QoL was 3 ± 1,5. De mediane follow-up was 68 (1-319) maanden waarin acht (1-51) ziekenhuiscontacten plaatsvonden. α-blokkers en 5-α-reductaseremmers werden gebruikt door 67 en 38% van de mannen. *Curatieve behandeling*. Van de 50 patiënten die met een curatieve intentie werden behandeld, kreeg 1 patiënt SNM en 49 patiënten (28%) prostaatdesobstructie. Hierna kon 33% stoppen met katheteriseren (OR 4,179). *Palliatieve behandeling.* Als eerste behandelstap werd gekozen voor CIC, CAD, SPC of watchful waiting (WW) in respectievelijk 20, 74, 6 en 0% van de gevallen. Als definitieve behandeling werd gekozen voor CIC, CAD, SPC en WW in respectievelijk 31, 21, 35 en 13% van de gevallen. Patiënten ondergingen een mediaan van drie behandelstappen (range 1-18) tot de definitieve behandeling werd bereikt. *Complicaties.* Katheterisatie geeft significant meer kans op een urineweginfectie (IRR 3,679; 95%-BI 2,920-4,686; *p* < 0,001) en macroscopische hematurie (IRR of 5,35; 95%-BI 2,292-15,12; *p* < 0,001) dan WW (zie tabel 7.1). Tussen katheterisatievormen geeft CIC significant minder complicaties dan CAD en SPC (p < 0,01), behalve voor hematurie. Een SPC geeft significant minder complicaties vergleken met een CAD (p < 0,05), behalve voor kathetergerelateerde pijn.

**Tabel 7.1:** Incidentie van complicaties per behandeloptie bij mannen met chronisch urineresidu. Data worden weergegeven als *incidence rate* per patiënt per jaar (95%-BI)

	**CIC**	**CAD**	**SPC**	**WW**
urineweginfecties	0,70 (0,62-0,80)	1,37 (1,160-1,62)	0,96 (0,84-1,09)	0,24 (0,19-0,30)
hematurie	0,06 (0,04-0,09)	0,17 (0,10-0,26)	0,08 (0,05-0,13)	0,02 (0,00-0,03)^a^
katheter problemen	0,04 (0,02-0,07)	0,30 (0,21-0,43)	0,11 (0,07-0,15)	0
katheter pijn	0,02 (0,01-0,04)	0,16 (0,09-0,25)	0,15 (0,11-0,21)	0
postrenale problemen	0	0	0,01 (0,00-0,04)^b^	0,12 (0,08-0,16)
blaasstenen	0,01 (0,00-0,02)^c^	0,01 (0,00-0,05)^d^	0	0,01 (0,00-0,02)^e^
urethra strictuur	0,01 (0,00-0,02)^c^	0	0	0,01 (0,00-0,02)^e^

*CAD* katheter à demeure, *CIC* intermitterende katheterisatie, *SPC* suprapubische katheter, *WW* watchful waiting.

a = 0,016 (0,006-0,034); b = 0,013 (0,003-0,036); c = 0,006 (0,001-0,020); d = 0,010 (0,001-0,048); e = 0,006 (0,001-0,021)

### Conclusie

Katheterisatie bij CUR kan gepaard gaan met complicaties. Een geselecteerde groep patiënten met een CUR zou voordeel kunnen hebben van een prostaatdesobstructie en daardoor kunnen stoppen met katheteriseren.

## 8. Hoeveel behandelstappen hebben OAB-patiënten nodig?

A.J. Seinen, R. Elburg, L.M. Hollegien, M.H. Blanker en L.P.W. Witte

Isala Ziekenhuis, Zwolle

### Introductie

In de dagelijkse praktijk kunnen de effectiviteit en de tolerantie van behandelingen voor overactieve blaas (OAB) per patiënt verschillen. Daarnaast duurt het vaak weken tot maanden voordat het effect van een behandeling kan worden beoordeeld. Dit leidt niet tot een snelle vermindering van klachten, maar juist tot een verminderde kwaliteit van leven en extra kosten. Het doel van dit onderzoek was inzicht te krijgen in de behandelingen die patiënten met OAB ondergaan, van diagnose tot uiteindelijke behandeling.

### Materiaal en methoden

Het betrof een *single-center*, retrospectieve cohortstudie van vrouwelijke patiënten, < 18 jaar, met de diagnose OAB. De inclusieperiode liep van 1 januari 2014 en 30 september 2016. De follow-up eindigde wanneer een patiënt een bevredigend behandeleffect ondervond en geen verdere behandeling nodig had, of op 1 januari 2020. De keuze voor een behandeling werd gemaakt door de patiënt en de uroloog samen. Het aantal, de volgorde en de duur van de aangeboden behandelstappen werden geanalyseerd.

### Resultaten

Er werden 120 patiënten geïncludeerd. De aangeboden behandelingen en de volgorde van behandelingen is weergeven in figuur 8.1. De behandeling begon meestal met medicatie, waaronder antimuscarinica (AM) (38%; 95%-BI 30-47). AM in combinatie met met bekkenbodemfysiotherapie (21%; 95%-BI 15-29) of mirabegron (11%; 95%-BI 6-18). Behandeling met botulinetoxine-A-injecties waren het meest effectief (67%; 95%-BI 42-85). Veelvoorkomende redenen voor het stoppen van een behandeling waren onvoldoende effect en bijwerkingen. Patiënten ondergingen een mediaan aantal behandelstappen van twee (1-6), met een mediane behandelingsduur van 28 weken (5-256).

**Figuur 8.1:**
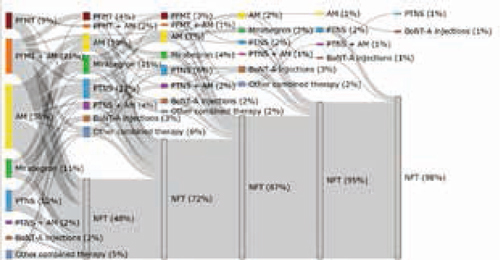
*Sankey plot* die de volgorde van behandelingen laat zien. *BoNT-A* botulinum toxin A, *PFMT* pelvic floor muscle therapy, *PTNS* percutaneous tibial nerve stimulation, *NFT* no further treatment

### Conclusie

De meeste patiënten proberen ten minste twee behandelingen voordat ze een bevredigende verlichting van OAB ervaren. Evaluaties van de behandeling vergen tijd, omdat de duur tot therapeutisch effect verschilt per patiënt en per behandeling. Onze bevindingen kunnen helpen bij het scheppen van reële verwachtingen voor patiënten bij het zoeken naar een behandeling voor OAB.

## 9. PSMA PET/CT bij patiënten met een biochemisch recidief prostaatkanker is geassocieerd met verbeterde oncologische uitkomsten na salvage radiatietherapie

D. Meijer, R.M. Mohede, W.S.C. Eppinga, B.G.L. Vanneste, P. Meijnen, O.W.M. Meijer, L.A. Daniels, R.C.N. van den Bergh, A.P. Lont, R.H. Ettema, F.H.K. Oudshoorn, P.J. van Leeuwen, H.G. van der Poel, M.L. Donswijk, D.E. Oprea-Lager, E.E. Schaake en A.N. Vis

Amsterdam Universitair Medisch Centrum, Amsterdam

### Introductie

De prostaatspecifiek membraanantigeen (PSMA) PET/CT heeft zijn diagnostische waarde voor het beter detecteren van ziektelokalisaties van prostaatkanker bij patiënten met een biochemisch recidief (BCR) na robotgeassisteerde radicale prostatectomie (RALP) inmiddels ruimschoots bewezen. De patiëntselectie die op dit moment nog lokale salvagebehandeling ondergaat, zoals salvage radiatietherapie (SRT), is daarmee ook veranderd. Hypothetisch gezien zouden patiënten die PSMA-geleide SRT ondergaan mogelijk betere oncologische uitkomsten hebben dan patiënten die ‘blinde’ SRT ondergaan. Het doel van deze studie was dan ook om de oncologische uitkomsten na SRT te vergelijken tussen patiënten die wel, en patiënten die geen PSMA PET/CT-scan hebben ondergaan voor BCR-prostaatkanker.

### Materiaal en methoden

Om deze oncologische uitkomsten te vergelijken tussen beide groepen, werd een historisch non-PSMA-cohort (2010-2015) vergeleken met een PSMA-cohort (2016-2020). Alle patiënten ondergingen SRT zonder hormonale therapie. De primaire uitkomstmaat was biochemische progressie een jaar na SRT, gedefinieerd als een PSA-waarde van 0,2 ng/ml of hoger boven de nadir na SRT. Om de uitkomsten beter te kunnen vergelijken, werd *case-control matching* toegepast.

### Resultaten

Van de 610 patiënten die werden geïncludeerd in deze studie, bleven er na het matchen nog 106 over in elk cohort, met een mediane PSA-waarde ten tijde van SRT van 0,3 ng/ml. In totaal hadden 30/212 patiënten (14%) biochemische progressie van de ziekte, een jaar na SRT. In het historisch cohort was dit 20% (21/106 patiënten) vergeleken met 8% (9/106 patiënten) in het PSMA-cohort (chi-kwadraattoets; *p* = 0,018).

### Conclusies

Patiënten die een PSMA PET/CT-scan ondergingen voor BCR-prostaatkanker hadden betere oncologische uitkomsten na SRT vergeleken met patiënten die ‘blinde’ SRT ondergingen. Concluderend, naast het toevoegen van waardevolle diagnostische informatie, is de PSMA PET/CT-scan nu ook geassocieerd met verbeterde oncologische uitkomsten voor patiënten met BCR na RALP.

**Tabel 9.1:** Karakteristieken van de geïncludeerde patiënten, na case-control matching

	**Alle patiënten** (*n* = 212)	**Historisch cohort** (*n* = 106)	**PSMA-cohort** (*n* = 106)	**p-waarde**
leeftijd bij SRT, jr; med. (IQR)	66 (62-71)	65 (61-69)	68 (64-72)	0,012
PSA-waarde bij SRT, ng/ml; med. (IQR)	0,3 (0,2-0,3)	0,3 (0,2-0,3)	0,3 (0,2-0,3)	1,0
totale toegediende dosis, Gy; mediaan (IQR)	70 (66-70)	66 (66-70)	70 (66-70)	0,001
pathologische Grade Group volgens ISUP; *u* (%)				
1	30 (14)	15 (14)	15 (14)	1,0
2	94 (44)	47 (44)	47 (44)	
3	46 (22)	23 (22)	23 (22)	
4	24 (11)	12 (11)	12 (11)	
5	18 (9)	9 (9)	9 (9)	
pathologisch T-stadium; n (%)				
≤ pT2	128 (60)	64 (60)	64 (60)	1,0
pT3a	64 (30)	32 (30)	32 (30)	
≥ pT3b	20 (10)	10 (10)	10 (10)	
snijvlakstatus; n (%)				
negatief	92 (43)	46 (43)	46 (43)	1,0
positief	120 (57)	60 (57)	60 (57)	
biochemische persistentie na RALP; *u* (%)				
nee	176 (83)	88 (83)	88 (83)	1,0
ja	36 (17)	18 (17)	18 (17)	
biochemische progressie 1 jaar na SRT; *u* (%)				
nee	182 (86)	85 (80)	97 (92)	0,018
ja	30 (14)	21 (20)	9 (8)	

*IQR* interquartile range, med. mediaan, *PSA* prostaatspecifiek antigen, *PSMA* prostaatspecifiek membraanantigeen, *RALP* robotgeassisteerde radicale prostatectomie, *SRT* salvage radiatietherapie, *SUP* International Society of Urological Pathology.

## 10. Strategieën om blaaskramp te voorkomen in de vroege periode na robotgeassisteerde radicale prostatectomie

H. Veerman, A. Houwink, P. Schutte, J.A. Nieuwenhuijzen, T.M. van der Sluis, T.A. Roeleveld, E. Wit, J.W. Mazel, A.N. Vis, P.J. van Leeuwen en H.G. van der Poel

Nederlands Kanker Instituut – Antoni van Leeuwenhoek, Amsterdam

### Introductie

Kathetergeïnduceerde blaaskrampen op de verkoeverkamer na robotgeassisteerde radicale prostatectomie (RARP) komen voor bij 60% van de patiënten. Het optimale behandelschema om blaaskrampen te voorkomen is onbekend.

### Materiaal en methoden

Er werd een prospectieve cohortanalyse uitgevoerd. Patiënten met bioptbewezen prostaatkanker, die waren behandeld met RARP tussen januari 2017 en april 2020 werden geïncludeerd. Combinaties werden vergeleken van algehele anesthesie en een *transverse abdominis plane* (TAP)-blok, met clonidine of ketamine, of een penisblok, of perivesicale infiltraties, en/of periurethrale infiltraties met ropivacaïne 20 ml 0,25%. De groepsgrootte (*n =* 42) was gepowered op 50% reductie van blaaskrampincidentie. Middels logistische regressie en *linear mixed models* werden verschillen tussen de blaaskrampincidentie (ja/nee), ernst (schaal 0-4) en algehele pijn (schaal 0-10) op de verkoeverkamer onderzocht.

### Resultaten

391 patiënten werden onderzocht in acht opeenvolgende cohorten. Een combinatie van TAP-blok, perivesicale en periurethrale injecties leidde tot de laagste incidentie van blaaskramp en vergeleken met het baselineprotocol (TAP-blok) leidde deze combinatie tot een daling van 49% (36% vs. 70%, *p* = 0,001). Ten opzichte van het baselineprotocol werd een lagere incidentie van blaaskrampen gevonden in de groep met TAP-blok en perivesicale injecties (46%, relatieve reductie 34%, *p* = 0,017) en de groep met TAP-blok en periurethrale injecties (52%, relatieve reductie 26%, *p* = 0,024). Er werden geen significante verschillen gevonden tussen alle protocollen met perivesicale en/of periurethrale injecties. Wanneer alle protocollen met perivesicale en/of periurethrale injecties werden vergeleken met alle protocollen zonder deze injecties, werd een reductie van 23% van de blaaskramp in de eerdere groep gevonden (45,6% vs. 60,7%; *p* = 0,001) Er werden geen verschillen in pijnscore gevonden tussen de protocollen. Zie figuur 10.1.

**Figure:**
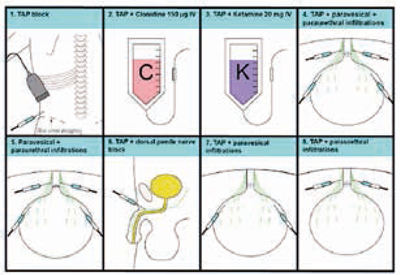
**Figuur 10.1**

### Conclusie

Perivesicale en periurethrale injecties met ropivacaïne 0,25% verminderen kathetergeïnduceerde blaaskrampen na RARP.

### 11. 3D-modellen gebaseerd op prostaat-MRI beïnvloeden de planning van zenuwsparing bij robotgeassisteerde radicale prostatectomie

H. Veerman, J.A. van der Eijk, J.H. Sluijter, T.N. Boellaard, C. Hoeks, T.A. Roeleveld, T.M. van der Sluis, J.A. Nieuwenhuijzen, E. Wit, E.J. Rijkhorst, M.J.A. van Alphen, R.L.P. van Veen, A.N. Vis, H.G. van der Poel en P.J. van Leeuwen

Nederlands Kanker Instituut – Antoni van Leeuwenhoek, Amsterdam

### Introductie

Op MRI gebaseerde driedimensionale (3D) beelden verbeteren het begrip van de precieze locatie van de tumor in de prostaat ten opzicht van tweedimensionale beelden. Deze studie onderzocht of op MRI gebaseerde 3D-prostaatmodellen de preoperatieve planning van zenuwsparing (ZS) bij robotgeassisteerde radicale prostatectomie (RARP) beïnvloeden.

### Materiaal en methoden

Van 20 patiënten met bioptbewezen, gelokaliseerd prostaatkanker (< cT3b) die een RARP hadden ondergaan werden 3D-modellen gemaakt. Een uro-radioloog teken-de de PC, het kapsel, de urethra en vesicula seminales in op axiale T2-gewogen 3 Tesla MRI-coupes. Hiervan werden virtuele en 3D-geprinte modellen gemaakt. Zeven RARP-urologen schatten de mate van mogelijke circumferentiële ZS per prostaathelft (0-6) in op basis van MRI, virtuele en 3D-prints. Een relevant verschil in planning tussen MRI en de 3D-modellen werd gedefinieerd als een verschil van ≥ 3. Middels de intraclass correlatiecoefficiënt (ICC) werd de overeenstemming in geschatte ZS tussen urologen onderzocht. Tevens werd de locatie van de indexlaesie (grootste laesie of hoogste Gleason-score) en de locatie van mogelijke extraprostatische extensie (EPE) volgens radiologische intekening vergeleken met het pathologieverslag. Zie figuur 10.1.

### Resultaten

Een relevant verschil in planning van ZS werd gevonden in 70/280 (25%) van de gevallen tussen MRI en virtuele 3D-modellen en in 73/280 (26%) van de gevallen tussen MRI en 3D-prints. De overeenstemming tussen urologen in geschatte ZS was hoger met de 3D-modellen dan met MRI (ICC MRI 0,40 (95%-BI 0,28-0,55); ICC virtuele modellen 0,52 (95%-BI 0,39-0,66); ICC 3D-prints 0,58 (95%-BI 0,45-0,71)). De locatie van de indexlaesie van de 3D-modellen en het radicale prostatectomiepreparaat kwam overeen bij 19/20 (95%) van de patiënten. De locatie van EPE kwam overeen bij 7/7 (100%) patiënten.

### Conclusie

3D-modellen zorgen voor beter inzicht in de tumorlokalisatie en planning van zenuwsparing bij RARP. De 3D-modellen veranderen de zenuwsparing in 1/4 van de gevallen en zorgen voor een hogere overeenstemming tussen urologen.

**Figuur 11.1:**
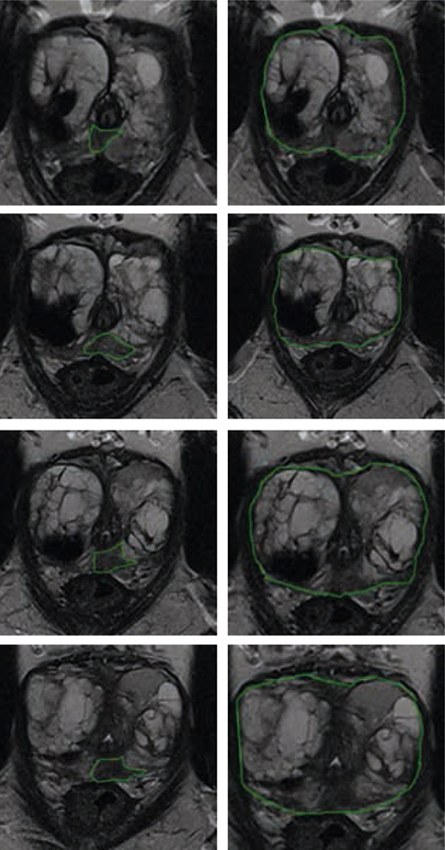
(Zie ook de volgende pagina.)

**Figuur 11.1:**
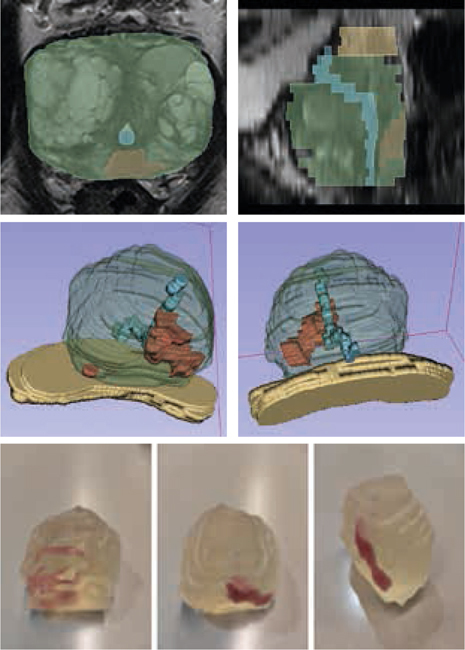
(Vervolg)

## 12. Validatie van confocale lasermicroscopie voor intraoperatieve snijvlakbeoordeling bij patiënten die een radicale prostatectomie ondergaan (ENCLOSURE-1)

D.J.H. Baas, W. Vreuls, V. Gorden, B. van Uijthoven, M. Wouters, J.P.M. Sedelaar, H.J.E.J. Vrijhof, R.J. Hoekstra, M.A.J. van Zanten, F. Mange, J.P.A. van Basten en D.M. Somford

Canisius Wilhelmina Ziekenhuis, Nijmegen

### Introductie

Radicale resectie bij mannen die een robotgeassisteerde radicale prostatectomie (RARP) ondergaan, is essentieel om het risico op een recidief te minimaliseren. Behoud van de neurovasculaire bundels (NVB) geeft een grotere kans op positieve snijvlakken, maar vergroot de kans op behoud van erectiele functie. In enkele centra wordt intraoperatieve beoordeling van de snijvlakken middels vriescoupe (NeuroSAFE) aangeboden. Het doel van dit onderzoek was te evalueren of intraoperatieve snijvlakbeoordeling middels confocale lasermicroscopie (CLM) een geschikt alternatief is voor NeuroSAFE.

### Materiaal en methoden

Tussen mei en augustus 21 werden 20 NeuroSAFE-patiënten gelijktijdig geëvalueerd middels CLM. De Histolog®-scanner werd gebruikt (SamanTree Medical SA, Lausanne, Zwitserland) om prostaatweefsel te scannen. Na de prostatectomie werden aan de posterolaterale zijde van de prostaat twee kapjes gesneden, na bewerking gescand met de Histolog®-scanner en beoordeeld op beeldkwaliteit door een getrainde pathologielaborant. Vervolgens werden de kapjes in 4-6 lamellen gesneden en ook gescand. De lamellen werden vervolgens geïnkt en verder verwerkt voor NeuroSAFE volgens protocol. De snijvlakken in vriescoupes werden als positief of negatief beoordeeld door een uro-patholoog. De CLM-beelden werden beoordeeld door één getrainde uropatholoog.

### Resultaten

Er werden 40 kapjes geanalyseerd. Drie patiënten hadden extracapsulaire extensie op MRI. Zes patiënten hadden een enkelzijdig positief snijvlak volgens NeuroSAFE. Vier van de zes NeuroSAFE-positieve patiënten waren ook positief bij CLM-beoordeling. Bij één patiënt kon het CLM-beeld niet worden beoordeeld vanwege cauterisatie-effect. Bij de andere patiënt was het prostaatweefsel niet volledig gescand. De mediane proceduretijd voor NeuroSAFE bedroeg 45 minuten en voor CLM 22 minuten.

### Conclusies

CLM is een veelbelovende techniek voor intraoperatieve snijvlakbeoordeling. Een gerandomiseerde studie is nodig om de niet-inferioriteit ten opzichte van intraoperatieve vriescoupes vast te stellen en om het langetermijneffect op de oncologische resultaten te evalueren.

## 13. De eerste klap is een daalder waard: maximale androgeendeprivatie met tweedegeneratie antiandrogenen onderdrukt het ontstaan van castratieresistent prostaatcarcinoom

J.M. Moll, W.J. Teubel, S.E. Erkens, A. Jozefzoon-Agai, N.F. Dits, A. van Rijswijk, W.M. van Weerden en G.W. Jenster Franciscus Gasthuis & Vlietland, Rotterdam

### Introductie

Maximale androgeenblokkade (MAB) met eerstegeneratie antiandrogenen (bicalutamide, flutamide) geeft bij uitgezaaid prostaatcarcinoom (PC) slechts een minimale winst ten opzichte van androgeendeprivatie (ADT) alleen. Tweedegeneratie antiandrogenen (apalutamide, enzalutamide) tonen wel een klinisch betekenisvolle meerwaarde. In deze studie hebben wij vier hormoongevoelige PC-modellen onderworpen aan ADT of MAB met eerste of tweedegeneratie antiandrogeen om verschillen in het ontstaan van CRPC en het biologisch gedrag bij progressie te bestuderen.

### Materiaal en methoden

4 AR positieve cellijnen (VCaP, DuCaP, PC346C en LAPC4) werden na verdeling over individuele kweekbakken blootgesteld aan ADT (*n =* 10) of MAB met bicalutamide (*n =* 10), flutamide (*n =* 10) of RD162 (*n =* 5), een analogon van enzalutamide en apalutamide. Karakterisering van CRPC-cellijnen vond plaats met behulp van AR-groeirespons door middel van blootstelling aan een titratiereeks androgeen met of zonder antiandrogenen en qPCR voor kritieke genen in de cascades van AR, AR-V7, hormoonproductie, glucocorticoïdreceptor, EMT en WNT.

### Resultaten

Het ontstaan van een CRPC-lijn verschilde per cellijn: VCaP 19/35, DuCaP 15/35, PC346C 34/35, LAPC4 15/35, *p* < 0,0001 en per behandeling: ADT 16/40, bicalutamide 32/40, flutamide 35/40, RD162 5/20, *p* < 0,0001. In PC346C maakte behandeling niet uit (*p =* 0,10). De AR groeirespons van CRPC verschilde per originele cellijn: VCaP was sterk AR-responsief, DuCaP was AR-hypersensitief, PC346C was zwak AR-responsief en LAPC4 was AR-onafhankelijk. Gestratificeerd naar originele cellijn, was er geen verschil in AR-groeirespons tussen CRPC na ADT of MAB. Expressie van SNAI1 en WNT5A is hoger bij onbehandelde PC346C en stijgt in de andere modellen bij het ontstaan van CRPC. In LAPC4 gaat de AR-cascade verloren.

### Conclusies

Zowel de eigenschappen van de primaire tumor als het gebruik van tweedegeneratie antiandrogenen zijn bepalend voor de kans op het ontstaan en het biologisch gedrag van CRPC. Resistentie tegen bicalutamide en flutamide ontstaat makkelijker dan tegen RD162. Er zijn echter geen verschillen gemeten in biologisch gedrag tussen ADT en MAB.

## 14. Het transprostatisch implantatiesysteem onder lokale anesthesie

O.P.J. Vrooman en M.R. van Balken

Rijnstate ziekenhuis Arnhem

### Introductie

De behandeling van benigne prostaathyperplasie met transprostatische implantaten is tot op heden veelal onder algehele anesthesie of sedatie toegepast. Met de toegenomen druk op OK-faciliteiten en om de behandeling maximaal minimaal invasief te maken, onderzochten we of deze behandeling ook onder lokale anesthesie goed kon worden uitgevoerd.

### Materiaal en methoden

Alle mannen die werden behandeld tussen 3 november 2020 en 1 juni 2021 werden geïncludeerd ter analyse van hun pijnbeleving tijdens de ingreep onder lokale anesthesie uitgevoerd in dagopname. Premedicatie betrof ciprofloxacine (500 mg), paracetamol (1000 mg), naproxen (250 mg) en midazolam 7,5 mg (< 70 jaar) of 3,75 mg (> 70 jaar). 20 minuten voor de start van de behandeling werd gekoelde (4 °C) chloorhexidine/lidocaïne gel (22 ml) ingebracht en met een penisklem werd de urethra afgesloten. Na afloop van iedere behandeling werd met behulp van een *Numeric Rating Scale* (NRS) met range 0-10 de pijnscore gemeten. Alle mannen die behandeld werden tot 20 april 2021 werden daarnaast geïncludeerd ter analyse van de verbetering ten aanzien van de initiële mictieklachten, zodat de resultaten na drie maanden follow-up beschikbaar waren. De behandelde mannen werden terug gezien na zes weken en drie maanden met een IPSS/QoL-score.

### Resultaten

Er werden 29 mannen behandeld. De leeftijd bedroeg 69,0 jr. ± 9,5 (mean ± SD). Het prostaatvolume bedroeg 37 ± 9,8 ml. Het aantal gebruikte implantaten was 3,1 ± 1,0. Bij vier mannen (14%) werd een katheter geplaatst na de procedure, 27 van de 29 mannen (93%) gingen dezelfde dag naar huis, twee bleven langer vanwege hematurie. De NRS van de 29 mannen bedroeg 3,8 (± 2,2). De verbetering van de IPSS-scores en toename van de QoL toonden ook onder lokale anesthesie een significante verbetering. Zie tabel 14.1.

**Tabel 14.1:** Functionele resultaten van transprostatische implantaten geplaatst onder lokale anesthesie

**Uitkomsten**	**6 weken**	**3 maanden**
**IPSS**		
*n* (totaal)	19	19
IPSS-baseline	24 ± 3,9	
IPSS-follow-up	13 ± 8,8	11 ± 7,2
IPSS-verandering	-11 ± 9,4	-13 ± 8,3
*p*-waarde	*p* < 0,0001	*p* < 0,0001
**QoL**		
*n* (totaal)	19	19
baseline	4,3 ± 1,0	
follow-up	2 ± 1,4	2 ± 1,6
verandering	-2 ± 1,9	-2 ± 1,9
*p*-waarde	*p* < 0,001	*p* < 0,001

### Conclusie

Ondanks dat er nog sprake is van een leercurve blijkt dat het goed mogelijk is om transprostatische implantaten te plaatsen onder lokale anesthesie. Dit maakt de kosten lager zonder dat dit ten koste gaat van de kwaliteit van de behandeling en er wordt minder gebruikgemaakt van schaarse operatiekamer en/of sedatiecapaciteit.

## 15. Retrospectieve analyse van preputiumplastieken bij jongens met pathologische phimosis, uitgevoerd gedurende de laatste negen jaar

J. Bosveld, P.A. Hornung, A.J. Klijn en R.P.J. Schroeder Universitair Medisch Centrum Utrecht, Utrecht

### Introductie

Phimosis is een aandoening van de voorhuid waarbij de voorhuid niet teruggetrokken kan worden. Bij de geboorte is dit een fysiologisch verschijnsel, maar op latere leeftijd kan phimosis pathologisch zijn. Traditioneel is besnijdenis, na topicaal corticosteroïdgebruik, de operationele behandeling van eerste keuze. De laatste jaren is er in verschillende landen een toenemende weerstand tegen het besnijden van kinderen. Bijgevolg is een toenemende tendens om in de plaats daarvan een preputiumplastiek uit te voeren. De huidige literatuur geeft geen uitsluitsel over welke operatieve benadering de voorkeur verdient. Daarom wordt in deze studie gekeken naar de langetermijneffecten van een preputiumplastiek bij kinderen met phimosis.

### Materiaal en methoden

Een retrospectieve cohortstudie werd uitgevoerd bij jongens jonger dan 18 jaar met phimosis, die in ons ziekenhuis een preputiumplastiek ondergingen tussen 1 januari 2011 en 1 januari 2020. De uitgevoerde preputiumplastiektechnieken bestonden uit een meervoudige Z-plastiek, een (meervoudige) Y-V-plastiek en een (meervoudige) *dorsal slit*. Kinderen die waren gediagnosticeerd met hypospadie of een begraven penis, evenals patiënten met een eerdere voorhuidoperatie werden geëxcludeerd. Het primaire resultaat van de preputiumplastiek was positief wanneer de voorhuid maanden na de operatie teruggetrokken kon worden.

### Resultaten

In totaal werden 176 patiënten in onze studie opgenomen. Er waren 40 patiënten met klachten van (recidiverende) balanitis. 62 van alle patiënten hadden een ernstige phimosis. 21 van hen werden gediagnosticeerd met lichen sclerose. 139 patiënten waren primair behandeld met topische corticosteroïden. Het resultaat van de preputiumplastiek was positief bij 163 patiënten (93%). Twee patiënten ontwikkelden wonddehiscentie en één patiënt kreeg een infectie na de uitgevoerde plastiek. Van de 13 patiënten (7%) met een negatieve uitkomst werd in acht gevallen een circumcisie verricht en in vier gevallen nogmaals een preputiumplastiek uitgevoerd.

### Conclusie

Preputiumplastiek is een haalbare operatieve behandeloptie bij pathologische phimosis.

## 16. Een preoperatief nomogram om operatieduur te voorspellen bij patiënten die een posterieure retroperitoneoscopische adrenalectomie ondergaan

A. van Uitert, E.C.J. van de Wiel, J. Ramjith, J. Deinum, H.J.L.M. Timmers, J.A. Witjes, L.J. Schultze Kool en J.F. Langenhuijsen Radboudumc, Nijmegen

### Introductie

Posterieure retroperitoneale adrenalectomie (PRA) heeft meerdere voordelen ten opzichte van de transabdominale laparoscopische benadering op het gebied van operatieduur, bloedverlies, postoperatieve pijn en herstel. Het kan echter een technisch uitdagende procedure zijn. Volgens de internationale consensus komen patiënten in aanmerking voor PRA als ze geopereerd moeten worden aan een goedaardige bijniertumor ≤ 7 cm en hun BMI < 35 kg/m^2^ is. Om de patiëntselectie verder te verbeteren, hebben we een preoperatief nomogram ontwikkeld om de operatieduur en complexiteit te voorspellen bij patiënten die een indicatie hebben voor PRA.

### Materiaal en methoden

Alle patiënten die een unilaterale PRA hebben ondergaan tussen februari 2011 en maart 2020 zijn geïncludeerd in de studie. Patiënten kwamen in aanmerking voor PRA als ze geopereerd moesten worden aan een goedaardige bijniertumor van ≤ 7 cm en de BMI < 35 kg/m^2^ was. De primaire uitkomstmaat was operatieduur als surrogaat voor chirurgische complexiteit. Door middel van 10 variabelen werd een predictiemodel gemaakt. Vervolgens werd door mid del van best-subsets regressieanalyse het beste één-variabeletot zeven-variabelenmodel gezocht.

### Resultaten

Er werden 215 patiënten geïncludeerd, met een gemiddelde leeftijd van 52 jaar en een gemiddelde tumorgrootte van 2,4 cm. Na de best-subsets regressieanalyse werd het vier-variabelenmodel geselecteerd en gekalibreerd, die de beste balans liet zien tussen voorspellende kracht en toepasbaarheid met een R^2^ van 38,6. In dit model werden geslacht, feochromocytoom, BMI en peri-nefrisch vet meegenomen, alle significante voorspellers voor de operatieduur.

### Conclusie

Om de preoperatieve patiëntselectie voor PRA te verbeteren, hebben we een vier-variabelennomogram ontwikkeld om de operatieduur te voorspellen. Als het nomogram een langere operatieduur voorspelt en dus een complexere operatie, zou de transabdominale benadering overwogen moeten worden, aangezien deze meer werkruimte geeft. Tevens kan het nomogram gebruikt worden voor trainingsdoeleinden om patiënten te selecteren met gunstige kenmerken voor urologen die PRA willen leren.

## 17. Overleving na neoadjuvante/inductie combinatie-immuuntherapie versus combinatie platinumchemotherapie voor lokaal gevorderd urotheelcarcinoom

S.M.H. Einerhand, N. van Dijk, J. van Dorp, J.M. de Feijter, M.L. van Montfoort, M.W. van de Kamp, T.N. Boellaard, K. Hendricksen, M.S. van der Heijden en B.W.G. van Rhijn Antoni van Leeuwenhoek – Nederlands Kanker Instituut, Amsterdam

### Introductie

De slechte uitkomsten van patiënten met stadium III-urotheelcarcinoom (d.w.z. cT3-4aN0M0 of cT1-4N+M0 UC), zelfs na neoadjuvante/inductie chemotherapie (NAIC), tonen het belang van effectievere systemische behandeling. We vergeleken de effectiviteit van NAIC met combinatie-immuuntherapie (ICI) bij patiënten die in ons instituut werden behandeld in 2018 en 2019.

### Materiaal en methoden

Data van patiënten die waren behandeld met NAIC werden verzameld uit onze prospectieve database. Patiënten die niet in aanmerking kwamen voor cisplatine of dit weigerden, werden behandeld met ICI (ipilimumab-nivolumab) in de NABUCCO-studie (NCT03387761; *n =* 24) of gemcitabine-carboplatin. Uitkomsten waren complete pathologische respons (pCR; ypT0N0), complete pathologische *downstaging* (pCD; ≤ ypT1N0), progressievrije overleving (d.w.z. progressie tijdens de behandeling of een recidief erna; PFS) en overleving.

### Resultaten

NAIC bestond uit een cisplatinegebaseerd regime (*n =* 73) of gemcitabine-carboplatin (*n =* 10). Patiënt- en tumorkarakteristieken – onder andere leeftijd, *Charlson Comorbidity Index*, ASA-score en nierfunctie – verschilden niet significant tussen de behandelgroepen. NAIC werd bij 11 patiënten (13%) vroegtijdig afgebroken vanwege progressie (*n =* 6) of toxiciteit (*n =* 5). ICI werd bij zes patiënten (25%) na twee cycli afgebroken vanwege toxiciteit (*p =* 0,205). Na NAIC ondergingen patiënten chirurgie (*n =* 50; 60%) of chemoradiatie (*n =* 26; 30%) en konden zeven patiënten (10%) geen consoliderende behandeling ondergaan vanwege progressie (*n =* 5) of toxiciteit (*n =* 2). Na ICI ondergingen alle patiënten chirurgie. Na chirurgie (*n =* 74) werd pCR door respectievelijk 11 (22%) NAIC- en 11 (48%) ICI-patiënten bereikt (*n =* 0,056). pCD werd door respectievelijk 17 (35%) NAIC- en 14 (58%) ICI-patiënten bereikt (*p =* 0,077). Patiënten die waren behandeld met NAIC kregen vaker progressie (43% vs. 8%; *p =* 0,001). Mediane (IQR) follow-up was 26 (20-32) maanden. In het gehele cohort (*n =* 107) was ICI geassocieerd met betere PFS (*p =* 0,003) en overleving (*p =* 0,003). Zie figuur 17.1.

**Figuur 17.1:**
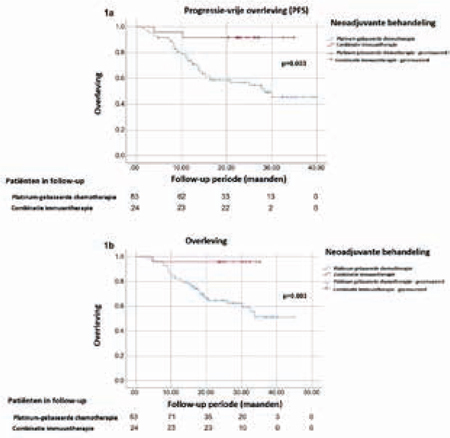
Betere progressievrije overleving (1a) en overleving (1b) van patiënten met stadium III-urotheelcarcinoom die zijn behandeld met combinatie-immuuntherapie vergeleken met platinumgebaseerde chemotherapie

### Conclusie

Uit onze data blijkt superieure overleving van patiënten met stadium III-UC die worden behandeld met neoadjuvante/inductie ICI vergeleken met NAIC.

## 18. Prostaatvolumemetingen in Nederland: MRI versus TRUS vergeleken met een prostatectomiepreparaat

G.W.B. Jansen, T. Arends, H. van Maanen, J.P. van Basten, R. Hoekstra, E. Vrijhof, M. Sedelaar en D.M. Somford Canisius Wilhelmina Ziekenhuis, Nijmegen

### Introductie

Het gebruik van multiparametrische MRI en *transrectal ultrasound of the prostate* (TRUS) om prostaatvolume te bepalen vergeleken met een prostatectomiepreparaat.

### Materiaal en methoden

Een prospectieve studie met patiënten die MRI en TRUS hadden ondergaan alvorens een robotgeassisteerde prostatectomie tussen januari 2020 en mei 2021 in Nederland. Prostaatmetingen werden verkregen door TRUS en MRI met de ellipsoïde formule. Resultaten werden vergeleken met het prostaat preparaat. Maximale interval tussen MRI, TRUS en prostatectomie was 6 maanden.

### Resultaten

214 patiënten werden geïncludeerd met een gemiddelde leeftijd van 66,2 jaar. Het gemiddelde prostaatvolume werd onderschat met zowel TRUS (53,4 cc) als MRI (57,6 cc) vergeleken met het prostaatpreparaat (68,3 g). Dit resulteerde in een 22% onderschatting en 16% onderschatting voor respectievelijk TRUS en MRI. Correlatie was goed met een Pearsons correlatiecoëfficiënt van 0,91 (TRUS) en 0,88 (MRI) en een *concordance correlation coefficient* (CCC) van 0,81 en 0,83. Regressieanalyse en Bland-Altman-analyse toonden acceptabele accuraatheid vergeleken met het prostaatpreparaat: R^2^ = 0,83 voor TRUS en R^2^ = 0,77 voor MRI. Metingen waren accurater in kleinere prostaten < 50 g: 31,2 cc (TRUS), 32,4 cc (MRI) en 40,9 g (preparaat) met 97% (TRUS) en 90% (MRI) van de metingen binnen 20 cc afwijking van het preparaat. Voor grotere prostaten, ≥ 50 g, waren gemiddelden 62,2 cc (TRUS), 67,8 cc (MRI) en 79,6 g (preparaat). Accuraatheid was minder met 58% (TRUS) en 69% (MRI) binnen 20 cc afwijking van het prostaatgewicht.

### Conclusie

Prostaatvolume gemeten door MRI en TRUS hebben vergelijkbare uitkomsten en een acceptabele voorspelling van het prostaatvolume. Zowel TRUS als MRI toonde onderschattingen van het prostaatvolume. MRI-metingen waren dichter bij het daadwerkelijke prostaatvolume, echter, het verschil met TRUS was klein. Beide modaliteiten kunnen ingezet worden en toegespitst worden op de voorkeur van de arts, beschikbaarheid en het lokale protocol of de voorkeur.

## 19. Robotgeassisteerde PSMA-radiogeleide chirurgie bij recidiverend prostaatkanker met de DROP-IN gammaprobe – een prospectief haalbaarheidsonderzoek

H.A. de Barros, M.L. Donswijk, M.N. van Oosterom, J.J.M.A. Hendrikx, F.W.B. van Leeuwen, H.G. van der Poel en P.J. van Leeuwen Antoni van Leeuwenhoek, Amsterdam, Prostaatkankernetwerk Nederland

### Introductie

Op prostaatspecifieke membraanantigeen (PSMA) gebaseerde radiogeleide chirurgie (RGC) is een veelbelovende techniek voor de detectie van prostaatkanker (PK)-laesies tijdens open salvagechirurgie. Toepassing van RGC bij robotgeassisteerde PK-chirurgie vraagt echter om nieuwe technologie. Doel van dit onderzoek was te evalueren of de geminiaturiseerde DROP-IN-gammaprobe robotgeassisteerde PSMA-RGC mogelijk maakt bij mannen met een recidief PK.

### Materiaal en methoden

In dit eerste prospectieve, in vivo haalbaarheidsonderzoek naar robotgeassisteerde RGC bij recidiverend PK werden 20 patiënten met ≤ 2 PK-recidieven in het kleine bekken (lokaal of lymfeklier) op PSMA PET/CT geïncludeerd (NCT03857113). Robotgeassisteerde PSMA-RGC met de DROP-IN-probe vond plaats 19-23 uur na intraveneuze toediening van een ^99m^Technetium-gelabeld PSMA ligand (^99m^Tc-PSMA-I&S). Primair werd de haalbaarheid van robotgeassisteerde PSMA-RGC onderzocht. Ook vond vergelijking van radioactiviteitmetingen en histologie plaats. Het PSA-gehalte werd 6-8 weken na chirurgie bepaald.

### Resultaten

Ten tijde van chirurgie bedroeg de mediane leeftijd 68 jaar (IQR 66-72) en was het mediane PSA 1,02 ng/ml (IQR 0,46-2,43). Met behulp van de DROP-IN-probe konden 19 van de 21 (90%) preoperatief geïdentificeerde laesies robotgeassisteerd gereseceerd worden met een mediane operatieduur van 128 min (IQR 103-157). Op laesieniveau bedroeg de sensitiviteit van PSMA-RGC 86% en de specificiteit 100%. Een PSA-daling > 50% en een PSA < 0,2 ng/ml werd waargenomen bij respectievelijk 12 van de 18 (67%) en drie van de 18 (17%) patiënten. Zie figuur 19.1.

**Figuur 19.1:**
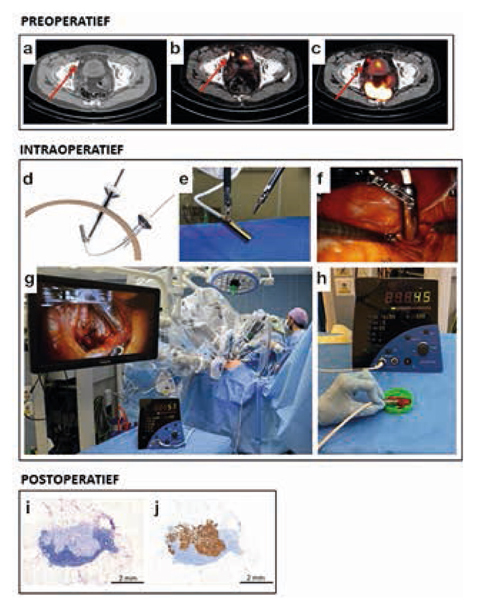
Robotgeassisteerde PSMA-RGC. Preoperatieve (**a**) CT, (**b**) PSMA PET/CT en (**c**) SPECT/CT tonen een prevesicaal PK-recidief (zie pijl). Met de DROP-IN-probe wordt in- en ex-vivoradioactiviteit van de verdachte laesie gemeten (**d-h**). Histologische analyse toont een PK-metastase aan in HE (**i**) en PSMA-kleuring (**j**)

### Conclusie

De DROP-IN-probe maakt robotgeassisteerde PSMA-RGC mogelijk. Middels deze procedure kunnen PK-laesies zeer specifiek gedetecteerd en gereseceerd worden.

## 20. Retrospectieve evaluatie van vroege urologische behandeling bij patiënten met spina bifida occulta

J.A. van der Leun, R.P.J. Schroeder, G. Tsachouridis, L. Hermsen en E. de Bruijn

Wilhelmina Kinderziekenhuis, Utrecht

### Introductie

Spina bifida occulta (SBO) is een aangeboren aandoening van de wervelkolom waarbij het neurale weefsel alleen bedekt is met huid. De ernst van urologische symptomen is hierbij zeer heterogeen. Het slechtste scenario is een neurogene blaas met detrusor-sfincterdyssynergie, wat kan leiden tot nier schade. Preventieve behandeling middels *clean intermittent catheterisation* (CIC), antimuscarinica en antibioticaprofylaxe is dan geïndiceerd. Momenteel is het onbekend hoeveel patiënten met SBO langdurige CIC nodig hebben. Het doel van deze studie was om dit aantal te identificeren. Daarnaast worden eventuele voorspellende factoren onderzocht.

### Materialen en methoden

Deze retrospectieve cohortstudie evalueert alle SBO-patiënten die waren behandeld in het Wilhelmina Kinderziekenhuis tussen 1990 en 2020. Inclusiecriteria waren: minderjarige (0-18) SBO-patiënten met minstens vier jaar follow-up. Alle patiënten van wie gegevens over de diagnose, de uitkomst of de initiële behandeling ontbraken, werden geëxcludeerd. Patiënten werden verdeeld in twee groepen op basis van de primaire uitkomst: wel of geen gebruik van CIC aan het eind van de follow-up. Baselinekenmerken en data van drie UDO’s werden uit het elektronisch medisch dossier verzameld en vergeleken. Analyse werd uitgevoerd met gebruik van de chi-kwadraattoets, de Fisher-exact-, de Mann-Whitney-U- of de T-test.

### Resultaten

Er werden 36 patiënten geïncludeerd. De mediane (IQR) leeftijd bij diagnose was 2,00 (0,23-13,13) maanden. De mediane (IQR) follow-up was 13,4 (8,34-17,83) jaar. Aan het einde van de follow-up gebruikten 13 patiënten (36,11%) CIC. Van de patiënten die geen CIC gebruikten, waren 11 patiënten (30,60%) nooit begonnen en 12 patienten (33,33%) gestopt. Op baseline waren er geen significante verschillen tussen de groepen. Het tweede UDO, bij een mediane (IQR) leeftijd van 29,27 (22,10-34,53) maanden, toonde een significant hoger mediaan postmictie residuvolume in de CIC-groep: 72,00 ml (42,5-135,00) vs. 11,00 ml (00,00-36,00).

### Conclusies

Ten minste een derde van alle SBO-patiënten heeft langdurige CIC-behandeling nodig. Daarnaast is er een significant hoger postmictieresidu in de CIC-groep.

## 21. De toepassing van 68Ga-PSMA-PET/CT bij patiënten die recent zijn gestart met active surveillance (PASPoRT-studie)

J.G. Heetman, P.D. Polm, T.F.W. Soeterik, J. Lavalaye, P.E.F. Stijns, L. Wever, H.H.E. van Melick en R.C.N. van den Bergh

St. Antonius Ziekenhuis, Nieuwegein

### Introductie

Patiëntselectie voor *active surveillance* (AS) blijft een uitdaging. Bij het gebruik van de huidige klinische parameters zoals MRI en gerichte biopten vindt er in 30-40% van de gevallen Gleason-upgrading plaats na prostatectomie. Het gebruik van 68GA-PSMA-PET/CT kan mogelijk lokale informatie toevoegen op moleculair niveau, en zo mogelijk de risico-inschatting verbeteren.

### Materiaal en methoden

De prospectieve cohortstudie PASPoRT (PSMA in Active Surveillance for PRostate Cancer Trial; NL69880.100.19) includeert patiënten met een diagnose prostaatkanker < 6 maanden met een indicatie voor AS. Alle deelnemers hebben een mpMRI ondergaan en vervolgens zijn er systematische en bij een zichtbare laesie, gericht biopten afgenomen. Vervolgens kregen alle patiënten een ^68^Ga-PSMA-PET/CT. Er werden aanvullende biopten afgenomen bij een PSMA-laesie (SUV_max_ > 4) indien die eerder niet zichtbaar was op de MRI en/of niet was gesampled met de systematische en/of gerichte biopten. De studie is gepowered om een upgrading van 10% te detecteren. Hiervoor zijn 141 patiënten nodig.

### Resultaten

Tot nu toe zijn er 86 patiënten geïncludeerd. De gemiddelde leeftijd is 67 jaar en het gemiddelde PSA is 6,66. Van alle patiënten hadden 17 (20%) een cT2 bij toucher en 5 (6%) een ISUP GG2. De gemiddelde SUV_max_ is 4,60. Aanvullende biopten werden bij 25 patiënten (29%) afgenomen. Van deze patiënten werden er 9 (10%) geüpgraded (6 GG2, 2 GG3, 1 GG5). De gemiddelde SUV_max_ van de geüpgrade groep is 7,81 en 5 patiënten hadden een PIRADS 1 of 2 op de mpMRI. In een multivariabele regressieanalyse zijn de SUV_max_ (*p =* 0,001) en een lage PIRADS-score (*p =* 0,009) geassocieerd met Gleason-upgrading. PSA, prostaatvolume en prostaatdensity hebben geen significant verband. In deze groep zijn op de PSMA PET/CT-scan geen metastasen gevonden.

### Conclusie

Onze voorlopige resultaten laten zien dat de toevoeging van PSMA-PET/CT, met aanvullende biopten van eerder niet zichtbare laesies, in potentie een toegevoegde waarde heeft bij patiënten met laagtot gemiddeld-risico prostaatkanker die eerder al een mpMRI hebben gehad, met name bij patiënten bij wie op de MRI geen afwijking zichtbaar was.

## 22. Verblijfsduur van dubbel-J-stents: een retrospectieve studie over risicofactoren voor post-ureterorenoscopie gecompliceerde urineweginfecties

L. Cools Paulino Pereira, A. Kums, P.M. Hennus en J. Beck Diakonessenhuis, Utrecht

### Introductie

Hoewel eerder is aangetoond dat het plaatsen van stents voorafgaand aan een ureterorenoscopie veilig is met weinig complicaties, wordt plaatsing geassocieerd met postoperatieve gecompliceerde urineweginfecties. Eerdere studies hebben de correlatie onderzocht tussen preoperatief geplaatste dubbel-J-stents en toename van bacteriële kolonisatie en bacteriurie; de associatie met toename van postoperatieve gecompliceerde urineweg-infecties blijft echter onduidelijk. Het doel van deze studie is te onderzoeken of preoperatieve dubbel-J-stents en de verblijfsduur van deze stents de kans op postoperatieve gecompliceerde urineweginfecties vergroten.

### Materiaal en methoden

Deze retrospectieve studie vond plaats in een ziekenhuis in Nederland. Alle volwassen patiënten die een ureterorenoscopie ondergingen in 2019 kwamen in aanmerking voor inclusie. Een deel van deze patiënten kreeg preoperatief een dubbel-J-stent, die vervolgens werd verwijderd of vervangen tijdens ureterorenoscopie. Gegevens over patiënten, verblijfsduur van de stents en de aanwezigheid van gecompliceerde urineweginfecties werden verzameld. Potentiële risicofactoren werden geëvalueerd middels univariate en multivariate logistische regressiemodellen.

### Resultaten

83 van de 195 geïncludeerde patiënten hadden preoperatief een dubbel-J-stent gekregen. 41,5% van alle patienten was vrouw; de mediane leeftijd was 56 jaar oud (IQR 38-67). 16,9% van de patiënten met een stent werd gediagnosticeerd met een postoperatieve gecompliceerde urineweginfectie, in vergelijking tot 7,1% in de groep zonder preoperatieve stent (*p =* 0,034). Echter, multivariate logistische regressieanalyse toonde identieke risicofactoren aan voor beide groepen, namelijk: vrouwelijk geslacht, preoperatieve positieve urinekweken en recidief van urolithiasis.

### Conclusie

Zowel dubbel-J-stents op zichzelf, als een langere verblijfsduur van dubbel-J-stents bleken beide geen significante risicofactoren te zijn voor postoperatieve gecompliceerde urineweginfecties. Vrouwelijk geslacht, preoperatieve positieve urinekweken en recidief van urolithiasis bleken wel significante risicofactoren.

## 23. De voorspellende waarde van urinecytologie bij de gradering van het urotheelcelcarcinoom van de hogere urinewegen (UTUC)

E. Cauffman, B. van der Heij en H.M. Bruins Zuyderland Medisch Centrum, Heerlen/Sittard

### Introductie

De keuze tussen een nefro-urectectomie (RNU) of niersparende chirurgie voor urotheelcarcinoom van de hogere urinewegen (UTUC) wordt gebaseerd op preoperatieve factoren, zoals spontane en selectieve urinecytologie en tumorgrootte. Literatuur over de voorspellende waarde hiervan is echter beperkt. Deze studie onderzoekt of deze variabelen bruikbaar zijn om de histologische uitkomst na nefro-ureterectomie te voorspellen en om te beoordelen of er vaker gekozen kan worden voor niersparende chirurgie.

### Materiaal en methoden

Alle patiënten die een RNU ondergingen voor UTUC tussen 2010 tot 2020 werden geïncludeerd. De urinecytologie werd geclassificeerd als benigne (TPS ≤ 3) of maligne (TPS 4 of 5) en vergeleken met de histologische bevindingen van de RNU, zijnde laaggradig (graad 1 en 2a) en hooggradig (graad 2b en 3) en stadiëring (pTis, pTa, pT1 en ≥ pT2).

### Resultaten

Van de 100 geïncludeerde RNU-preparaten bleek 65% hooggradig en 46% spierinvasief. Maligne cytologie had een sensitiviteit en specificiteit voor hooggradig UTUC van respectievelijk 60 en 69% en voor spierinvasief UTUC van 67 en 65%. De positief en negatief voorspellende waarde was resp. 78 en 48% voor hooggradig UTUC en 62 en 70% voor spierinvasief UTUC. De gemiddelde tumorgrootte was 4,2 cm bij patiënten met een hooggradige UTUC en 3,6 cm bij een laaggradige UTUC. Een grotere tumor (per centimeter) had een odds ratio van 1,35 (95%-BI 1,06-1,71) voor hooggradig UTUC en 1,16 (95%-BI 0,921-,45) voor spierinvasief UTUC.

### Conclusie

Urinecytologie heeft een matige voorspellende waarde voor gradering en invasiviteit van UTUC. Bij patiënten met maligne cytologie was in 40% van de gevallen sprake van een laaggradig UTUC en in 33% van niet-spierinvasief UTUC. Tumorgrootte was geassocieerd met tumorgradering, maar niet met tumorstadiëring. Met de huidige criteria van de *European Association of Urology* worden mogelijk kansen gemist om meer patiënten niersparend te behandelen. Deze data kan een argument zijn om in selecte gevallen toch te kiezen voor niersparende behandeling, ondanks de aanwezigheid van hooggradige cytologie of een tumor > 2 cm.

## 24. Evaluatie van de schildwachtklierbiopsie bij peniskanker in een perifeer ziekenhuis

A.M.T.J. Heemels, M.R. van Balken, P.C. Weijerman, R. Koot, B. Hendrikx en B.K. Kroon

Rijnstate Ziekenhuis, Arnhem

### Introductie

Sinds 2014 worden patiënten met peniskanker in ons ziekenhuis behandeld vanuit het streven zorg waar mogelijk dichtbij de patiënt te organiseren. Een essentieel onderdeel van deze behandeling is de schildwachtklierbiopsie. Hierbij kunnen occulte metastasen in cN0-liezen worden opgespoord. Het doel van deze studie was om de resultaten van de schildwachtklierbiopsie in ons ziekenhuis te evalueren.

### Materiaal en methoden

Vanaf 2014 tot 2021 werden 94 opeenvolgende patiënten met een hoog stadium (> T1aG1) peniscarcinoom prospectief in deze studie geïncludeerd. De gemiddelde leeftijd was 71 jaar (41-99). 88 patiënten hadden beiderzijds klinisch onverdachte liezen (cN0). Zes patiënten hadden enkelzijdig een onverdachte lies en contralateraal een klierpositieve lies (cN1). Schildwachtklierbiopsie werd verricht in de 182 onverdachte liezen. Behandeling van de primaire tumor werd voorafgaand of in dezelfde sessie verricht. Preoperatief werd een lymfoscintigram gemaakt na injectie met 99mTechnetium-nanoclloïd rondom de tumor of het litteken. De schildwachtklier werd intraoperatief geïdentificeerd met behulp van een blauwe speurstof en een gammaprobe. Alleen bij een tumorpositieve schildwachtklier werd later een liesklierdissectie uitgevoerd.

### Resultaten

De mediane follow-up was 23 maanden (2-81). Lymfoscintigrafie visualiseerde ten minste 1 schildwachtklier bij 93 patiënten (99% detectie). Er werden 219 schildwachtklieren gevisualiseerd en 238 verwijderd. De schildwachtklier bleek positief bij 13 patiënten (15 liezen). Complicaties (alle Clavien 1 of 2) traden op bij 19% (18/94) van de geopereerde patiënten. Bij één patiënt werd na een negatieve schildwachtklierprocedure een tumorpositieve klier in de lies aangetroffen na een follow-up van vijf jaar. Dit resulteerde in een foutnegatief percentage van 7% (1/14). Dit is lager dan gerapporteerd wordt in de literatuur (meta-analyse:12%).

### Conclusie

Schildwachtklierbiopsie bij peniskanker in ons ziekenhuis verloopt succesvol en is veilig: het detectiepercentage is hoog, de sensitiviteit is hoog en de morbiditeit is acceptabel.

## 25. Clomifeencitraat voor verbetering spermakwaliteit bij subfertiele mannen

M. Huijben, M.T.W.T. Lock, V.F. de Kemp, L.M.O. de Kort en H.M.K. van Breda

Universitair Medisch Centrum Utrecht

### Introductie

Subfertiliteit bij mannen is een veel voorkomend en wereldwijd probleem. Clomifeencitraat (CC) is een selectieve oestrogeenreceptorblokker en kan de spermakwaliteit verbeteren door de hormoonsynthese en spermatogenese te stimuleren. Er is gebrek aan bewijs over de werkzaamheid van CC als therapie voor mannelijke subfertiliteit. Het doel van deze studie was de effectiviteit en veiligheid van CC voor subfertiele mannen te onderzoeken.

### Materiaal en methoden

In deze *single-center* studie zijn mannen die met CC werden behandeld vanwege subfertiliteit retrospectief geanalyseerd. De primaire uitkomst was verandering in semenparameters. Secundaire uitkomstenmaten waren evaluatie van totaal testosteron (TT), luteïniserend hormoon (LH) en follikelstimulerend hormoon (FSH), zwangerschap, hemoglobine (Hb), hematocriet (Ht), prostaat specifiek antigeen (PSA), bijwerkingen, potentiele voorspellers voor biochemische en/of klinische respons.

### Resultaten

In totaal werden 52 subfertiele mannen behandeld met CC. Bij 6/52 patiënten vermeldde de anamnese testosterongebruik of misbruik (2 op medische indicatie). Bij 13/46 patiënten (28%) zonder testosterongebruik in de voorgeschiedenis was er sprake van een verbetering van zaadcelconcentratie, bij 22 patiënten (48%) van de totale zaadcelmotiliteit en bij 20 patiënten (43%) een verbetering van de VCM (volume x progressieve concentratie x motiliteit). Bij 7/13 patiënten met verbetering in spermaconcentratie (54%) kwam een zwangerschap tot stand (spontaan of met behulp van inseminatie). Bij alle zes mannen met testosterongebruik in de voorgeschiedenis trad een verbetering van semenkwaliteit op. TT-, FSH- en LH-waarden stegen tijdens behandeling (*p <* 0,05). Er waren geen veranderingen in Hb, Ht en PSA tijdens de behandeling. Bij 8% van de patiënten traden milde bijwerkingen op tijdens CC-behandeling (agitatie, transpiratie). Laagnormaal FSH vóór CC-behandeling was voorspellend voor semenverbetering tijdens behandeling.

### Conclusie

CC lijkt een veilige therapie die bij 43% van de subfertiele mannen een verbetering in VCM laat zien, zonder significante bijwerkingen.

## 26. Clomifeencitraat: een alternatief voor testosterontherapie bij hypogonadale mannen met een kinderwens

M. Huijben, M.T.W.T. Lock, V.F. de Kemp, J.J.H. Beck, L.M.O. de Kort en H.M.K. van Breda

Universitair Medisch Centrum Utrecht

### Introductie

Hypogonadisme is een wereldwijd probleem bij mannen dat seksuele, fysieke en mentale problemen veroorzaakt. Testosterontherapie is behandeling van eerste keuze voor mannelijk hypogonadisme, met diverse bijwerkingen, zoals subfertiliteit. Clomifeencitraat (CC) is een alternatieve *off-label* behandeling voor hypogonadale mannen, vooral voor mannen met een actieve of toekomstige kinderwens.

Er is gebrek aan studies over het gebruik van CC voor mannen met hypogonadisme. Het doel van deze retrospectieve studie was om de effectiviteit en veiligheid van CC voor hypogonadale mannen te evalueren.

### Materiaal en methoden

In deze *single-center* studie werden mannen die werden behandeld met CC voor hypogonadisme retrospectief geevalueerd. De primaire uitkomst waren hormonale parameters, inclusief totaal testosteron (TT), vrij testosteron (FT), luteïniserend hormoon (LH) en follikelstimulerend hormoon (FSH). Secundaire uitkomstmaten waren hypogonadale symptomen, metabole en lipidenparameters, hemoglobine (Hb), hematocriet (Ht), prostaatspecifiek antigeen (PSA), bijwerkingen en mogelijke voorspellers voor de biochemische en/of klinische respons.

### Resultaten

In totaal werden 153 hypogonadale mannen behandeld met CC. De mediane behandelduur was 10 maanden (range 1-96). De gemiddelde TT-, FT-, LH- en FSH-waarden stegen tijdens de behandeling. TT steeg van 9 naar 16 nmol/l (zie figuur 26.1), met een biochemische respons bij totaal 89% van de patiënten. Gestegen TT-waarden hielden aan tot en met acht jaar na behandeling. Met CC-therapie had 74% van de patiënten een verbetering van hypogonadale symptomen. Laagnormaal LH vóór CC-behandeling was voorspellend voor een betere TT-respons. Tijdens CC-therapie werden weinig bijwerkingen gemeld en werden geen klinisch belangrijke veranderingen in PSA, Hb en Ht gevonden. Zie figuur 26.1.

**Figuur 26.1:**
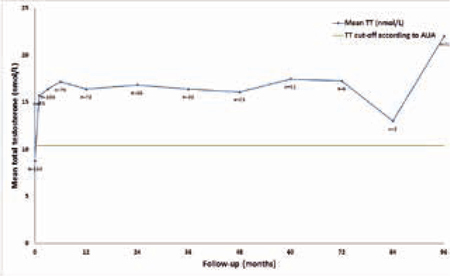
Langetermijnresultaten van clomifeencitraat op totaal testosteron

### Conclusie

CC is een effectieve therapie op korte en lange termijn, die zowel klinische symptomen als biochemische parameters van mannelijk hypogonadisme verbetert met minimale bijwerkingen en goede veiligheidsaspecten.

## 27. Evaluatie van de resultaten en de leercurve van robotgeassisteerde radicale cystectomie voor blaaskanker

J.G.R.W. Kleuskens, S. Motmans, C. Berendsen, T. Tuytten, R. Bos, L. Driessen en H.M. Bruins

Zuyderland Medisch Centrum, locatie Heerlen en Sittard/Geleen

### Introductie

In het laatste decennium is er een evidente toename van het aantal robotgeassisteerde radicale cystectomieën (RARC), waarbij slechts beperkte informatie bekend is over de impact van de leercurve op perioperatieve uitkomsten. In deze studie werden de resultaten sinds de introductie van de RARC in ons ziekenhuis geëvalueerd.

### Materiaal en methoden

Alle patiënten die tussen februari 2014 en april 2021 een RARC voor blaaskanker ondergingen, werden geïncludeerd. Patiënten ondergingen een in opzet intracorporele urinedeviatie (ICUD) en werden geopereerd door in totaal vier urologen. Primaire uitkomstmaten waren de 30- en 90-dagen-mortaliteit (30dM/90dM), heropnamepercentage (30dH/90dH) en complicatiepercentage volgens Clavien-Dindo (30dC/90dC). Secundaire uitkomstmaten waren snijvlakstatus, operatieduur (OT), bloedverlies (EBL), opnameduur (LoS) en ziektespecifieke overleving (DSS).

### Resultaten

177 patiënten werden geïncludeerd. De 30dM en 90dM was 1,1% en 5,1%. Het percentage complicaties > 2 volgens Clavien-Dindo was 24,3% zowel na 30 als 90 dagen. De 30dH en 90dH was 22,6% en 23,7%, met minder heropnames in 2019 en 2020. 12 van de 173 ICUD’s moesten worden geconverteerd. Na 2019 werd deze conversie niet meer gezien. Positief snijvlakpercentage was 2,6%. Het mediaan EBL was 400 ml (IQR 250-700), mediane OT was 450 min (IQR 411-490), mediane LoS was 9 dagen (IQR 7-12) en mediane lymfeklieropbrengst was 15 (IQR 11-21). DSS was 31,6% bij een mediane follow-up van 20 maanden. Er werd een afname gezien van de mediane OT van 520 naar 420 minuten. Overige secundaire uitkomstmaten verbeterden minimaal of bleven gelijk bij het doorlopen van de leercurve.

### Conclusie

De oncologische en perioperatieve resultaten van RARC zijn grotendeels in lijn met de resultaten van grote open radicale cystectomieseries. De 30dC, maar niet de 90dC, lijkt wat hoger te zijn. Verder is de operatieduur langer in vergelijking met de open radicale cystectomie, maar deze nam wel af tijdens de studieperiode. Een evidente impact van de leercurve op de resultaten anders dan operatieduur werd niet geobjectiveerd.

## 28. Klinische waarde van de ADXBLADDER-test in de follow-up van hooggradig niet spierinvasief urotheelcelcarcinoom van de blaas

A.E. van der West, D. van de Kerkhof, E.L. Koldewijn en T.J.N. Hermans

Catharina Ziekenhuis, Eindhoven

### Introductie

De urine-MCM5-analyse (ADXBLADDER) zou kunnen worden ingezet in de follow-up van het niet-spierinvasief urotheelcelcarcinoom van de blaas (NMIBC). Wij delen de eerste resultaten uit onze praktijk aangaande patiënten in de follow-up van hooggradig (HG) NMIBC.

### Materiaal en methoden

Gedurende zes maanden zijn in één centrum 43 patiënten in de follow-up van HG NMIBC geselecteerd middels controle van spreekuren. Patiënten werden geïncludeerd binnen de eerste drie jaar follow-up. Behandelbeleid werd enkel gebaseerd op cystoscopie én cytologisch urineonderzoek. Er werd extra urine verzameld voor de ADX-BLADDER-test. Bij klinische verdenking op een recidief werd zo nodig een transurethrale resectie tumor (TURT) verricht. Exclusiecriteria waren een katheterisatie en/of urineweginfectie binnen twee weken voor de ADXBLADDER-test.

### Resultaten

De primaire pathologie bestond uit: pTaHG (*n =* 7), pT1HG (*n =* 26), CIS (*n =* 6) en CIS + pTa/1HG (*n =* 4). Bij 34 patiënten werd een re-TURT verricht (alle pT1 tumoren). Alle patiënten kregen een adjuvante behandeling middels chemospoelingen (*n =* 4), BCG-spoelingen (*n =* 31) of BCG- en chemospoelingen (*n =* 8). De gemiddelde follow-up na diagnose was 14 maanden. 10 patiënten hadden een positieve ADXBLADDER, waarvan twee klinisch de verdenking hadden op een recidief blaastumor (positief-voorspellende waarde 20%). Hierbij was er een verdenking op persisterend CIS bij cystoscopie en positieve urinecytologie (TPS 5). Omdat er geen klinische consequenties waren, werd er geen TURT verricht (= limitatie). 33 patiënten hadden een negatieve ADXBLADDER, waarvan er drie klinisch de verdenking hadden op een recidief blaastumor. Echter, TURT toonde geen carcinoom (negatief-voorspellende waarde 100%). Het lage recidiefpercentage in dit specifieke HG NIMBC-cohort beperkt echter vooralsnog de power van de voorspellende waarden. Zie tabel 28.1.

**Table:** **Tabel 28.1**

		**HG NIMBC (urinecytologie/cystoscopie/PA)**		
		**aanwezig**	**afwezig**	
ADXBLADDER	positief	2	8	10
*(n = 43)*	negatief	0	33	33
		2	41	

*8* sensitiviteit 2/2 = 100%, *9* specificiteit 33/41 = 81%, *: PPV* = 2/10 = 20%,; *NPV* = 33/33 = 100%.

### Conclusies

In dit cohortonderzoek had 77% (33/43) van de cystoscopieën gereduceerd kunnen worden middels ADXBLADDER. De beperkte power noodzaakt echter tot verdere validatie in een groter HG NMIBC-cohort.

## 29. Levensverwachting van de Nederlandse uroloog: verontrustende cijfers

B.K. Kroon, M.R. van Balken en H.M. Kroon

Rijnstate Arnhem

### Introductie

In 2016 en 2019 is onderzoek verricht naar de levensverwachting van algemeen chirurgen in Nederland. Deze bleek duidelijk lager dan de gemiddelde levensverwachting van andere hoogopgeleide Nederlanders en ook lager dan die van de gemiddelde Nederlandse medisch-specialist. In deze studie onderzoeken we de levensverwachting van de Nederlandse uroloog.

### Materiaal en methoden

Via de NVU werden geboorte- en sterftedata opgevraagd.

De gemiddelde sterfteleeftijd werd berekend. Daarnaast werd de gemiddelde sterfteleeftijd berekend van urologen die hun pensioengerechtelijke leeftijd haalden. Deze cijfers zijn vergeleken met de online beschikbare cijfers van het CBS en verstrekte cijfers van de SPMS.

### Resultaten

Sinds 2010 worden sterftecijfers door de NVU bijgehouden. Op basis van 58 overleden urologen was de gemiddelde levensduur 75,1 jaar (47,3-98,6). De urologen die hun pensioen haalden (*n =* 44) werden gemiddeld 81,3 jaar oud (65,8-98,6). De levensverwachting van de Nederlandse uroloog is 5,1 jaar korten dan van de gemiddelde Nederlandse man (80,2). In vergelijking met de hoger opgeleide Nederlandse man is de levensverwachting van de Nederlandse uroloog 9,1 jaar korter (84,2 vs. 75,1) en 6,1 jaar korter dan van de hoger opgeleide man die de leeftijd van 65 bereikt heeft (87,4 vs. 81,3). Urologen worden zelfs nog minder oud dan chirurgen: 75,1 vs. 78,1. Gepensioneerde urologen worden gemiddeld 6,6 jaar minder oud dan hun gepensioneerde collega medisch-specialisten (81,3 vs. 87,9). Verdere uitsplitsing van de cijfers per medisch specialisme werd door de SPMS niet verstrekt.

### Conclusies

De levensverwachting van Nederlandse urologen is duidelijk lager dan die van andere Nederlanders, hoogopgeleide Nederlanders en medisch-specialisten in het algemeen. Urologen zijn dus net als algemeen chirurgen weinig duurzame medisch-specialisten. Een vervolgstap stap naar mogelijke oorzaken en uiteindelijk verbetering van deze verontrustende uitkomsten zou een vergelijkbare analyse per specialisme zijn om de levensverwachting voor elk specialisme in kaart te brengen.

